# Induction of Plant Resistance in Tobacco *(Nicotiana tabacum)* against Tomato Spotted Wilt Orthotospovirus through Foliar Application of dsRNA

**DOI:** 10.3390/v13040662

**Published:** 2021-04-12

**Authors:** Naga Charan Konakalla, Sudeep Bag, Anushi Suwaneththiya Deraniyagala, Albert K. Culbreath, Hanu R. Pappu

**Affiliations:** 1 Department of Plant Pathology, University of Georgia, Tifton, GA 31793, USA; nagacharan.konakalla@slu.se (N.C.K.); Anushi.Deraniyagala@uga.edu (A.S.D.); spotwilt@uga.edu (A.K.C.); 2 Department of Plant Protection Biology, Swedish University of Agricultural Sciences, 230 53 Alnarp, Sweden; 3 Department of Plant Pathology, Washington State University, Pullman, WA 99163, USA; hrp@wsu.edu

**Keywords:** double-stranded RNA, tomato spotted wilt virus, N gene, NSs gene, viral suppressors of RNA silencing, RNA interference, vsiRNA, virus resistance

## Abstract

Thrips-transmitted tomato spotted wilt orthotospovirus (TSWV) continues to be a constraint to peanut, pepper, tobacco, and tomato production in Georgia and elsewhere. TSWV is being managed by an integrated disease management strategy that includes a combination of cultural practices, vector management, and growing virus-resistant varieties where available. We used a non-transgenic strategy to induce RNA interference (RNAi)-mediated resistance in tobacco (*Nicotiana tabacum*) plants against TSWV. Double-stranded RNA (dsRNA) molecules for the NSs (silencing suppressor) and N (nucleoprotein) genes were produced by a two-step PCR approach followed by in vitro transcription. When topically applied to tobacco leaves, both molecules elicited a resistance response. Host response to the treatments was measured by determining the time to symptom expression, and the level of resistance by absolute quantification of the virus. We also show the systemic movement of dsRNA_N from the inoculated leaves to younger, non-inoculated leaves. Post-application, viral siRNAs were detected for up to nine days in inoculated leaves and up to six days in non-inoculated leaves. The topical application of dsRNAs to induce RNAi represents an environmentally safe and efficient way to manage TSWV in tobacco crops and could be applicable to other TSWV-susceptible crops.

## 1. Introduction

Tomato spotted wilt disease (TSWD) in the USA was detected in 1974 in peanut [1], and field epidemics were reported in Texas by 1985 [2]. The disease is caused by the tomato spotted wilt virus (TSWV; *Tospoviridae*; genus *Orthotospovirus*) and is vectored by several species of thrips. The two main thrips species that efficiently transmit TSWV are tobacco thrips (*Frankliniella fusca* (Hinds)) and western flower thrips (*F. occidentalis* (Pergande)), and both are common in the southeastern USA [3]. The broad host range of both TSWV and thrips vectors makes disease management difficult and requires the implementation of multiple tactics in an integrated fashion [4]. TSWV was first confirmed in flue-cured tobacco in Georgia in 1986, and since then the virus has become a major constraint to the production of several crops including pepper, tobacco, tomato, and peanut in Georgia [5,6].

The severity of tomato spotted wilt epidemics and resulting damage in tobacco varies from year to year. Tobacco remains an economically important crop in Georgia even though the acreage has decreased over the years, from 7300 hectares in 2010 to 2800 hectares in 2020. While the reduction is partly due to supply and demand, TSWV is also a major factor as a 10% stand loss early in the season is attributable to TSWV with 20–35% of final incidence of symptomatic plants each year [7]. That resulted in an 18 percent loss in yield with an economic loss of over $9.5 million in Georgia alone [7]. In Georgia and other tobacco-growing states in the southeastern US, growers are reducing the tobacco acreage due to spotted wilt disease and non-profitability.

Considerable progress was made toward developing an integrated disease management program for TSWV in peanut, where growing TSWV resistant or tolerant varieties is the central component. A Risk Index for TSWV based on production and pest management practices was developed which was adapted widely by Georgia growers resulting in the reduced impact of TSWV in peanut [7,8]. While the use of biologics that trigger systemic acquired resistance was shown to be effective against TSWV in tobacco [9,10], there are, however, no known tobacco varieties with resistance to TSWV, and current management practices almost exclusively depend on thrips management [11,12,13].

A relatively recent addition to the toolbox for developing resistant varieties includes RNAi-based virus resistance, which offers a promising approach as part of this general strategy [14]. RNAi was used to develop transgenic plants with desirable traits, particularly for the control of viral diseases [15,16]. However, the acceptance of the cultivation of transgenic crops has not been realized worldwide [17,18,19,20,21]. One approach to alleviate the biosafety issues of transgenic plants is the use of non-transgenic, RNAi-derived virus resistance. Resistance could be induced through topical/foliar application of dsRNA molecules, as it has been shown that dsRNA is the crucial molecule in the induction of RNAi-derived resistance, and a topical application in the form of a spray was found to be effective in triggering resistance against several viruses [22,23].

dsRNA molecules that are homologous to a particular mRNA sequence induce the endogenous RNA silencing or RNA interference (RNAi) mechanism via a sequence-specific degradation [24,25,26,27]. RNA silencing plays a significant role in plant defense against plant viruses [28,29,30,31,32,33,34]. Studies have shown that dsRNAs and small interfering RNAs (siRNAs) are the key players that initiate RNAi [35]. There are several reviews that summarized the state of the art of RNAi [36,37,38,39]. Previous studies have shown that exogenous application of dsRNA molecules produced either in vitro or in vivo methods by targeting the viral silencing suppressor sequences conferred resistance against several cognate plant viruses such as bean common mosaic virus (BCMV) [29], papaya ringspot virus (PRSV) [40], pigeonpea sterility mosaic virus (PPSMV) [41], sesbania mosaic virus (SeMV) [42], tobacco mosaic virus (TMV) [43,44], and zucchini yellow mosaic virus (ZYMV) [45].

Methods to produce long (up to 4 kb) dsRNA followed by large-scale production include in vitro hybridization of sense and antisense single RNA strands [46]. These were synthesized by T7 DNA-dependent RNA polymerase combined with either in vitro RNA transcription catalyzed by the RNA-dependent RNA polymerase or by in vivo RNA amplification employing phi6 polymerase complexes in a phi6 bacterial cell line carrier [47,48,49,50]. Other strategies include the use of bacterial expression systems such as bacteriophage λ dependent *red* recombination, which were effective for dsRNA production [43].

While plants use RNAi as a defense mechanism, plant viruses code for viral suppressors of RNA silencing (VSRs) to counter the host’s RNAi. VSRs of different viruses were shown to use other mechanisms to overcome the host’s defense [14]. TSWV genome consists of three single-stranded RNAs designated as L (8.9 kb), M (4.8 kb), and S (2.9 kb). The L RNA is completely in a negative sense and contains a gene encoding the L protein (RdRP). The ambisense M RNA encodes the NSm protein (movement protein) and the structural proteins, G1 and G2 [51]. The ambisense S RNA encodes N (nucleocapsid) protein and NSs protein (RNA silencing suppressor) [52]. The NSs protein of TSWV acts as a VSR and mainly localizes and aggregates in paracrystalline arrays in the cytoplasm in plant and insect cells [53].

The aim of this study was to develop a sprayable biological molecule to induce plant resistance against TSWV in tobacco. Toward that goal, we used a two-step PCR to incorporate a T7 RNA polymerase promoter sequence at the 5’ ends of NSs and N genes. The in vitro produced dsRNAs were exogenously applied to tobacco leaves and were tested for their efficacy to trigger resistance to TSWV. We also determined the accumulation of N gene-targeted dsRNA molecules, as well as siRNAs in the younger, non-inoculated leaves, using stem-loop PCR [54], and investigated the movement of dsRNAs and vsiRNAs from inoculated leaves (where dsRNA applied) to younger, non-inoculated leaves.

## 2. Materials and Methods

### 2.1. Nicotiana tabacum cv. NC 196 Growth Conditions

*Nicotiana tabacum* cv. NC 196 is commercially grown in Georgia and several other states in the southeastern USA and was used in this study. Plants were grown in a growth chamber with 25/22 °C day/night temperature and 16/8 h light/dark cycles. Ten tobacco plants, at the four-leaf stage, were used in each treatment, and each experiment was repeated three times. TSWV isolate used in this study were obtained from naturally infected tobacco and tomato crops grown in the UGA Farms and were maintained on *N. tabacum* plants in a growth chamber.

### 2.2. Targeting TSWV Genome Sequences for dsRNA Production

Sequences of N and NSs genes were obtained from NCBI GenBank, and a multiple alignment tool [55] was used to identify the most conserved regions within each gene. Consensus sequences of each gene were then used for designing primers using the Primer 3 selection tool (Table 1) [56].

### 2.3. Two-Step PCR Approach and In Vitro Transcription

For the production of dsRNA molecules targeting the N and NSs genes, an in vitro method based on a two-step PCR approach was used as previously described [34,37]. A T7 RNA polymerase promoter sequence was introduced at both 5’ and 3’ ends in the second PCR reaction using a specific T7 linker primer (Table 1). PCR conditions were 95 °C 2 min, 95 °C 30 s, 58 °C 30 s, 72 °C 30 s, and 72 °C 5 min for 35 cycles for the N-gene and 95 °C 2 min, 95 °C 30 s, 55 °C 30 s, 72 °C 30 s, and 72 °C 5 min for 35 cycles for the NSs-gene; each reaction mixture was comprised of 2 µL 10× Thermo Taq polymerase buffer, 2 µL of the 1st PCR product, 0.08 µL of Thermo Taq DNA polymerase, 0.4 µL dNTPs (10 mM), 1.6 µL volume of T7 linker primer (10 pm/µL), and the final volume was adjusted to 20 µL with water. The second PCR product was used as a template for in vitro transcription and production of the respective dsRNA.

The resulting construct/2nd PCR product was used in an in vitro transcription system, T7 Ribomax^TM^ Express large-scale RNA production kit (Promega, Madison, WI, USA). The reaction mixture comprised of 10 µL Ribomax^TM^ Express T7 2X buffer, 3 µL of N or NSs amplicons, 2 µL enzyme mix, and the final volume of 20 µL was adjusted with water. The reaction mixture was maintained at 37 °C for 4 h, 85 °C for 10 min, and 25 °C for 20 min. The concentration of total nucleic acid molecules was estimated by NanoDrop™ One/OneC Microvolume UV-Vis Spectrophotometer (Thermo Fisher Scientific, Waltham, MA, USA).

### 2.4. Topical Application of N and NSs dsRNA on Tobacco Leaves

Inoculum mixture was prepared by adding 4 µL of in vitro produced dsRNA (N or NSs) to 16 µL of TSWV-infected plant sap (4 × 10^5^ dilution) which was obtained by using inoculation buffer (0.1 M phosphate buffer (pH 7.0) and 0.01 M 2-mercaptoethanol) mixed with 1% of silicon carbide (320 grit). The sap was extracted from 20 days post-inoculation (dpi) TSWV-infected tobacco plants and per 300 mg of tissue, 600 µL of inoculation buffer was used. Each plant received 250.2 µg of dsRNA_N and 280.5 µg of dsRNA_NSs. The positive control group consisted of inoculating two leaves with TSWV sap at 4 × 10^5^ dilution, and the negative control group consisted of 0.1 M phosphate buffer (pH 7.0) and 0.01 M 2-mercaptoethanol-treated tobacco leaves. Ten plants per treatment were inoculated by gently rubbing 20 µL of the inoculation mixtures onto two fully expanded leaves dusted with silicon carbide. Inoculated leaves were thoroughly washed with 0.05% Triton X-100 in 10 min intervals for one-hour post-inoculation (hpi) and received a final wash with water. Inoculated plants were maintained in the conditions mentioned above, and symptom development was monitored until 20 dpi.

### 2.5. Stability of dsRNA and Transport in Nicotiana tabacum

To monitor the stability and transport of dsRNA in plants over time, 4 µL (250.2 µg) of dsRNA targeted to the N gene was mixed with 16 µL water, and the mixture was applied to two fully expanded silicon carbide-dusted *N. tabacum* leaves by gently rubbing. The dsRNA-treated tobacco leaves were washed with Triton X-100 (0.05%) and water, and treated plants were kept in the growth chamber.

Two leaf disks (~5 mm) from inoculated, as well as non-inoculated leaves, were collected from all plants at time points one hpi, three, six, and nine dpi. Leaf samples from 10 individual plants were pooled and total RNA was extracted using TRIzol (Invitrogen, Carlsbad, CA, USA) and RNA concentration was determined as mentioned above. cDNA was synthesized by using gene-specific TSWV N-R primer (2 pm/µL) and 50 ng total RNA. Primers TSWV N-F and TSWV N-R were used to detect the presence of dsRNA_N in the inoculated and non-inoculated leaves.

### 2.6. RT-qPCR Assay for the Detection of Viral RNA Copy Number in Inoculated and Non-Inoculated Leaves

Total plant RNA was extracted from inoculated plants using TRIzol at three, six, 12, and 20 dpi. cDNA was synthesized using 100 ng of total RNA in a 10 µL reaction volume employing SuperScript III reverse transcriptase (Invitrogen, Carlsbad, CA, US. cDNA was diluted two-fold with water before performing qPCR. Each qPCR reaction consisted of 2 µL of diluted cDNA, 6 µL of SSo Advanced Universal SYBR Green Supermix (Bio-Rad, Hercules, CA, USA), and 1 µL of each primer (10 µM) [45] (Table 1) and performed in a CFX 96 well qPCR thermal cycler (Bio-Rad, Hercules, CA, USA). The reaction was carried out under the following conditions: 95 °C for 3 min, followed by 35 cycles of 95 °C for 15 s, 55 °C for 45 s, and 72 °C for 15 s. At the end of each annealing step, the fluorescent signal of SYBR Green was measured. The cycle thresholds (Ct) were calculated by CFX Maestro Software (Bio-Rad, Hercules, CA, USA). For the purpose of absolute quantification, the TSWV partial N gene sequence was cloned into the pGEM-T Easy vector (Promega, Madison, WI, USA) used as a standard for quantitative analyses. Conversion of double-stranded DNA (dsDNA) to nanograms was performed considering the average molecular weight of a base pair of deoxynucleotide (650 Da) and the recombinant DNA base pair number. Avogadro’s constant (6.022 × 10^23^ molecules/mol) was used to estimate the dsDNA copy number. To generate external standard curves for absolute quantification, 10-fold serial dilutions containing circular dsDNA at 10^9^ to 10^0^ molecules/µL of each plasmid were prepared and analyzed by qPCR with the two designed primers [57] (Table 1). Total cDNA from healthy plants of tobacco were run in parallel to ensure the absence of any TSWV amplification. Each sample collected at different time points as mentioned above was analyzed in triplicate, and the mean quantification cycle (Cq) and its SE were calculated. Every Cq mean value was extrapolated on the corresponding standard curve. TSWV copy number was calculated from the formula 10^(Cq−b/slope of the standard curve)^, where b is the y-intercept.

### 2.7. Detection of TSWV siRNA by Stem-Loop RT-PCR

The 21-nt long TSWV siRNA hotspot in the N gene was used to identify a consensus vsiRNA of TSWV. In brief, the TSWV hotspot used in the study was located within the antisense strand of 717 bp fragment of the N gene of TSWV (used for the production of dsRNA) [47]. RT reaction was done with siRNA3S_RT primer (1 µM), and SuperScript III reverse transcriptase using the conditions of 16 °C 30 min, 30 °C 30 s, 42 °C 30 s, 50 °C 1 s for 60 cycles, and 85 °C 5 min according to a pulsed RT protocol [54]. The targeted vsiRNA was amplified using siRNA3S_F (10 µM) as the forward primer and universal reverse primer (10 µM), using the PCR conditions 94 °C 2 min, 94 °C 15 s, and 60 °C 1 min. Primer sequences are shown in Table 1.

### 2.8. Statistical Analysis

The data obtained were statistically analyzed by using Statistica v 8.1 (StatSoft, Round Rock, TX, USA) and MS office excel software. To investigate the effect of dsRNA and TMV on tobacco by using contrast statistics, a student’s t-test was performed and two-tailed *p* values are reported throughout. Differences at *p* ≤ 0.05 were considered significant and represented with different letters.

## 3. Results

### 3.1. Production for dsRNA for TSWV N and NSs Genes

**Figure 1:**
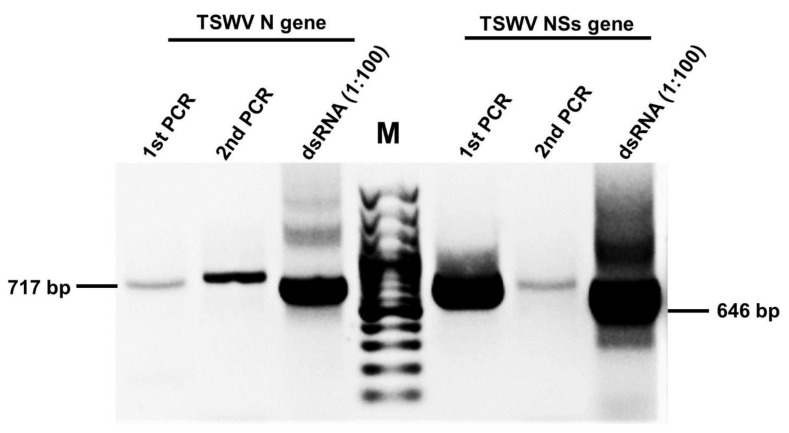
Production of dsRNA molecules specific to the tomato spotted wilt virus nucleocapsid (N) gene and non-structural (NSs) genes by two-step PCR method and in vitro transcription. M: 100 bp DNA ladder.

A 717 bp fragment within the N gene and a 646 bp fragment within the NSs gene whose sequences were highly conserved among the known TSWV isolates were selected. Both fragments were amplified using primers that contained a linker sequence at their 5’ end. In the second PCR reaction, a T7-linker primer sequentially introduced a T7 promoter sequence at the 5’ ends of both sense and antisense strands of the targeted DNA fragments. The T7 Ribomax^TM^ Express large-scale RNA production system was used to transcribe the second PCR product. The products of the first PCR, second PCR, and the in vitro transcription reaction for the N and NSs genes are shown in Figure 1.

### 3.2. dsRNA-Dedicated Resistance in Tobacco against TSWV

**Figure 2:**
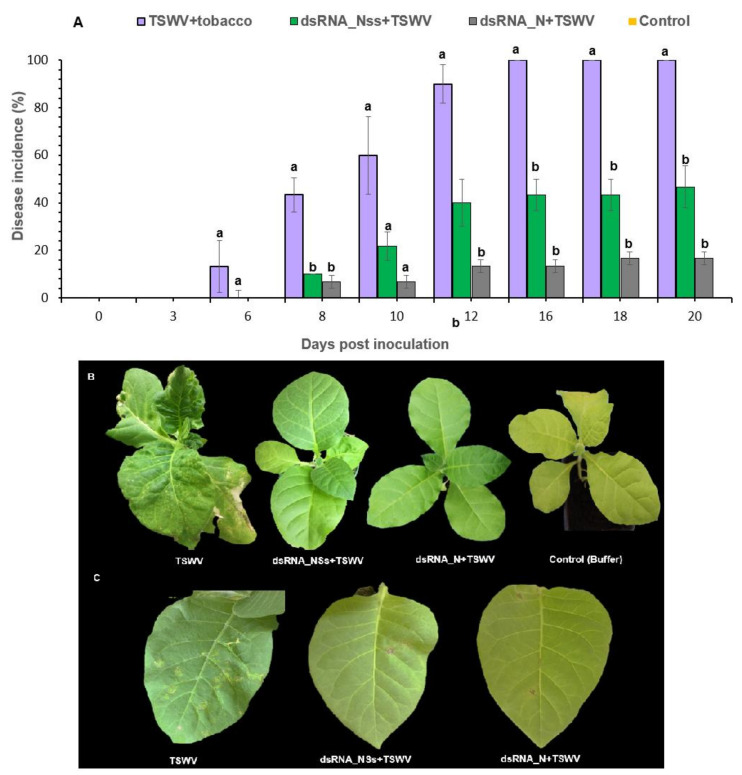
Effect of double-stranded RNA on tomato spotted wilt virus (TSWV) infection in tobacco. (**A**) TSWV + tobacco; dsRNA_N + TSWV; dsRNA_Nss + TSWV and Control (Buffer). Disease incidence was calculated based on the number of plants exhibiting systemic disease symptoms out of 10 plants used in each treatment. Results were expressed as mean values of three independent experiments. Error bars represent standard error. Different letters indicate significant differences between groups (*p* ≤ 0.05) (**B**) Tobacco plants that received dsRNAs + TSWV exhibited no disease phenotype, (**C**) Effect of dsRNAs on lesion formation on tobacco leaves. Leaves of infected plans in dsRNA treatments had fewer lesions compared to TSWV treatment alone.

In order to test the efficacy of the dsRNA molecules in suppressing TSWV infection, ten *N. tabacum* plants were used for each of the following treatments: (a) TSWV plus dsRNA_N, (b) TSWV plus dsRNA_NSs, (c) TSWV (positive control), and (d) inoculation buffer (negative control). At the sap dilution of 5 × 10^2^, TSWV-inoculated plants (positive control to ensure that the TSWV inoculum is infectious), exhibited systemic vein clearing and chlorotic lesions and necrosis at six dpi in inoculated leaves, and all plants exhibited severe mosaic symptoms at 12 dpi (Figure 2B). Symptom development was monitored in all treatments until 20 dpi. From the three experiments performed, based on symptom appearance and subsequent progression, 84% of the plants in the dsRNA_N + TSWV treatment had no visible symptoms, whereas 54% of dsRNA_NSs + TSWV-treated plants did not develop any symptoms (Figure 2A). Both treatments protected tobacco plants from TSWV infection, compared to 100% of the plants that were inoculated with TSWV only became symptomatic. Plants that received dsRNAs topically (either dsRNA_N and dsRNA_NSs) exhibited a delay in symptom expression since no symptoms were observed at six dpi, whereas at the same time point, 13% of TSWV-inoculated plants exhibited severe TSWV disease symptoms (Figure 2A,B).

**Figure 3:**
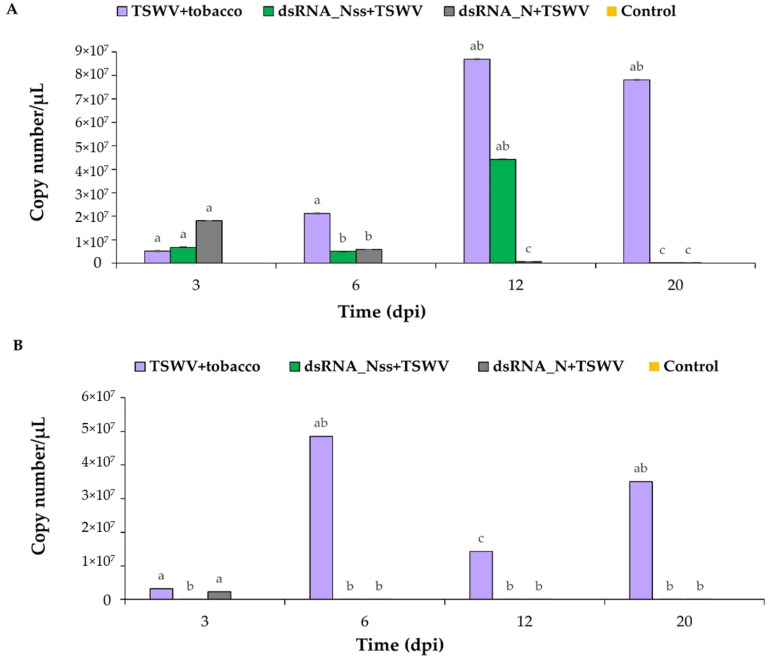
Accumulation of tomato spotted wilt virus (TSWV) in inoculated and younger, non-inoculated leaves of tobacco at different time points. Logarithmic graph of the viral copy numbers of four different treatments (dsRNA_N + TSWV, dsRNA_NSs + TSWV, TSWV+ tobacco, and control. (**A**) TSWV copy number in co-inoculated leaves and (**B**) TSWV copy number in younger, non-inoculated leaves. Different letters indicate statistically significant differences (*p* < 0.05) between means. Error bars represent standard error.

The copy number of TSWV was determined by employing qRT-PCR and the presence of vsiRNAs was confirmed by using stem-loop RT-PCR, respectively [54,57]. In the positive control plants (those inoculated with TSWV only), a constant increase in TSWV copy number (=accumulation) was noticed from three dpi, which was reached its maximum at 12 dpi in the TSWV-inoculated plants (Figure 3A). In the dsRNA_N + TSWV treatment, the TSWV accumulation was significantly lower from six dpi in the inoculated leaves. Although the dsRNA_NSs + TSWV treatment showed significantly less accumulation at six dpi in the inoculated leaves, the virus copy number was increased at 12 dpi (Figure 3A) in this treatment. In the non-inoculated/young leaves of the dsRNA_N + TSWV treatment, the TSWV accumulation was significantly lower than the younger leaves of TSWV treatment (Figure 3B) from 3 dpi, whereas dsRNA_NSs + TSWV treatment showed a significant difference from 6 dpi onwards. (Figure 3B).

### 3.3. Systemic Movement of TSWV dsRNA_N

**Figure 4:**
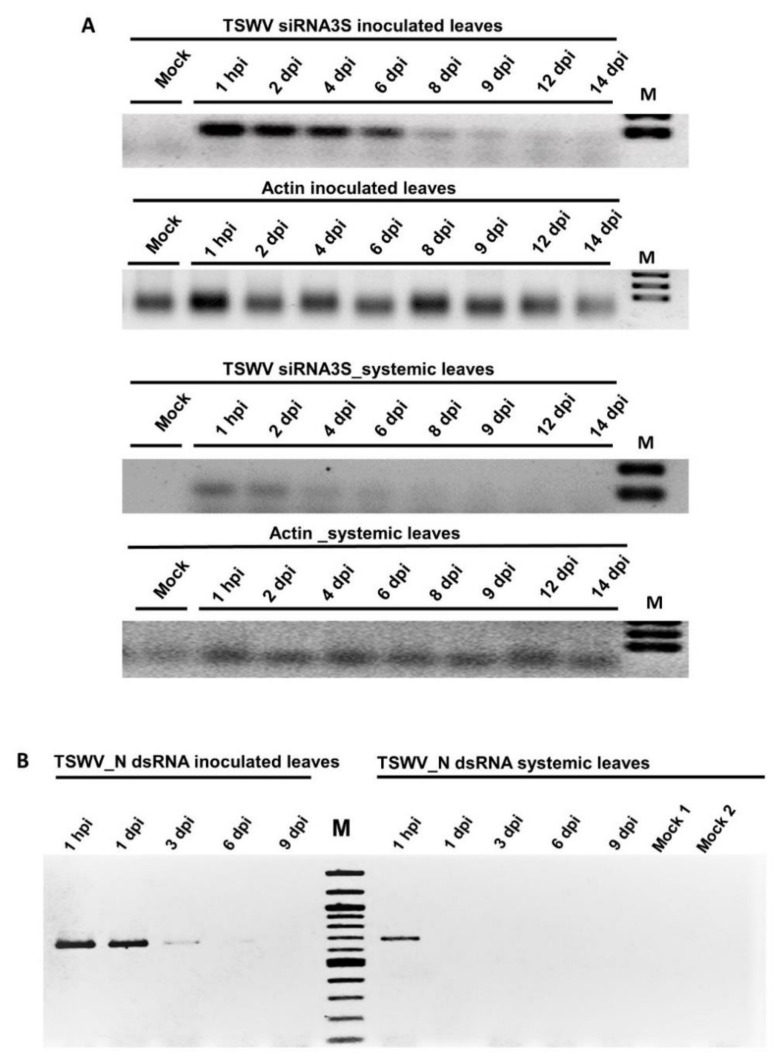
Movement of tomato spotted wilt virus (TSWV) dsRNA_N and siRNA3S in tobacco (**A**) Accumulation of siRNA3S from 1 hpi to 14 dpi. M: 100 bpt DNA ladder. (**B**) Detection of the TSWV_N dsRNA in inoculated and non-inoculated leaves at different time points. Mock 1 is mock-inoculated leaves, Mock 2 is younger, non-inoculated leaves from the same Mock 1 plants.

To determine if the dsRNA produced against the N gene could move systemically in tobacco, its presence over time was determined by semi-quantitative RT-PCR. RT-PCR results showed that the N gene dsRNA levels in the inoculated leaves continuously declined from six dpi, at nine dpi the signal was very low in agarose gel and was non-detectable at 12 dpi. Interestingly, dsRNA_N was detected in the non-inoculated leaves beginning from one hpi and no bands were detected afterward (Figure 4B). This experiment showed the movement of dsRNA from the inoculated to younger, non-inoculated leaves (Figure 4B).

### 3.4. Detection of a siRNA of N Gene by Stem-Loop RT-PCR

The presence of siRNA3S was monitored in the dsRNA-inoculated and non-inoculated leaves by stem-loop RT-PCR [45]. The putative siRNA, resolved as a specific band of 60 bp, was found beginning from one-day post dsRNA application and lasted up to nine dpi in inoculated leaves and six dpi in non-inoculated leaves. No amplification was seen in the inoculated leaves of plants treated with water (Mock, Figure 4A).

## 4. Discussion

TSWV continues to be a production constraint to several agronomic and horticultural crops in the USA and other parts of the world [15]. Resistant varieties offer the most cost-effective and environmentally friendly option for reducing the impact of this virus. RNAi was shown to be the mechanism behind host plant resistance to virus infection [14]. Transgenic technology has been used to induce RNAi in plants to attain pathogen-derived resistance against TSWV by targeting the N and NSm genes [58,59,60,61,62,63]. Due to the public concerns about the biosafety issues surrounding genetically modified crops, search for alternate, non-transgenic approaches for inducing RNAi for virus resistance has been the subject of research for the last several years. Here we showed that exogenously applied dsRNA targeted to the N and NSs genes of TSWV triggered RNAi in tobacco plants and the treated plants showed resistance to the virus. The strategy has several advantages compared to the use of transgenic plants. Firstly, this approach is faster since plant transformation, regeneration, and subsequent characterization of transgenic events are not needed. Secondly, dsRNAs for various genes could be prepared in a relatively short period of time, and their efficacy could be tested in a ‘shotgun approach’. Thirdly, mixed viral infections could be managed by introducing dsRNAs for multiple viruses at once. Furthermore, this strategy could target both vectors (such as thrips) and plant viruses simultaneously [64]. It has been shown that RNA-based vaccination was effective against a bipartite geminivirus and where a single dsRNA molecule could protect against two tomato-infecting viruses, tomato yellow leaf curl virus and cucumber mosaic virus [65].

The dsRNA molecules produced in vitro for N and NSs genes of TSWV, when topically applied on tobacco leaves, proved to be effective against TSWV infection. Interestingly, a recent study by Tabein et al. (2020) [66] used a similar strategy against TSWV in *N. benthamina* and emphasized that the choice of the target viral sequence in designing RNAi-based vaccines is crucial for its success. The observed differences in antiviral efficiency of the N- and NSs-targeting dsRNAs could be due to the biological role of the N and NSs gene products. In our study, dsRNA_N provided a higher level of resistance against TSWV when compared to that of the dsRNA_NSs. NSs sequesters the siRNAs resulting in the inhibition of the formation of replication initiation and silencing complex (RISC) [67]. It is possible that the exogenously applied dsRNA targeted to NSs inactivated the silencing suppressor, and thereby facilitating the spread of viral siRNAs. Successful establishment of TSWV infection requires the replication and transcription of the viral genes to produce infectious ribonucleocapsid proteins (RNPs), the minimal infectious unit containing the three genomic RNAs that are tightly packed by the N protein and few copies of the viral RdRp. In this context, the degradation of the N-coding small RNA by RNAi could impact viral accumulation due to the central role of the N protein, which was manifested in a higher level of resistance induced by a dsRNA targeted to the N gene. There are similar findings of the greater efficacy of dsRNAs targeting the capsid coding genes and silencing suppressor genes in the case of several other viruses [68].

There is a decrease in lesion size on tobacco leaves that were inoculated with either dsRNA_N or dsRNA_NSs compared to TSWV-inoculated plants (Figure 2B). This may indicate that the vsiRNAs produced from the exogenously applied dsRNA by endogenous RNAi machinery of tobacco are reducing or interfering with virus infection in inoculated leaves.

The systemic transport and stability of dsRNA molecules produced against TSWV genes in tobacco were studied to explore other possible means of dsRNA application (i.e., drenching) or clay nanosheets/Bio Clay [29,69]. dsRNA_N, upon application to the leaves, was quickly transported to the systemic leaves at one hpi. The movement of dsRNA might be similar to that of viroids. Viroids do not encode any proteins and are not encapsidated but utilize the host cellular factors for their spread throughout the plant [70]. The existing hypothesis about long-distance RNA transport is that once an RNA molecule enters the phloem sieve tube, it moves from the source to sink organs by simple diffusion [71]. The dsRNA used in this study may contain some motifs which could interact with cellular proteins facilitating their transport. Several previous reports described the systemic movement of dsRNA in plants [44,72,73,74,75]; however, topically applied dsRNAs remained mostly in treated leaves in watermelon and squash plants [45], or in tomato [73]. The differences in these studies could be explained by the existence of an active dsRNA long-distance movement process mediated by proteins binding to specific RNA motifs [75]. In contrast, Northern blot studies by Tenllado and Díaz-Ruíz (2001) [76] did not reveal the presence of dsRNA in the non-inoculated leaves when dsRNA was exogenously applied against pepper mild mottle virus in *N. tabacum*. A possible explanation for this difference could be the lower sensitivity of Northern blot assays relative to semi-quantitative and real-time RT-PCR used in the present study.

Finally, the accumulation of vsiRNAs in plants treated with dsRNA_N suggests that the RNAi processed the externally applied N dsRNA. SiRNA3S was detected in the treated leaf (where dsRNA was applied) and in the non-treated leaf at one hpi. The latter indicates either that siRNAs produced in the dsRNA-treated leaf are transported rapidly to non-inoculated leaves or that they are produced in the non-inoculated leaves from the rapidly transported dsRNA. Further studies on the successful application/delivery of dsRNA vaccine at field conditions as well as preventive and curative experiments to demonstrate the effectiveness of TSWV dsRNA on tobacco remains to be carried out.

In conclusion, we demonstrated that topical application of dsRNAs derived from the N and NSs genes conferred resistance to TSWV in tobacco. It remains to be seen if the resistance will be equally effective in other TSWV-affected crops such as peanut, pepper, and tomato.

## Figures and Tables

**Table 1:** Primers for RT-PCR amplification of TSWV N and NSs genes, qRT-PCR and stem-loop RT-PCR.

Primer	Oligonucleotide Sequence (5′-3′)	Target Gene	Amplicon Size (bp)
TSWV N-F	GTCTAAGGTTAAGCTCACTAA	Nucleocapsid (N)	717
TSWV N-R	AAGAGTTTCACTGTAATGTTC		
TSWV NSs-F	AGTCTGGGGATCAACTGCATC	Nonstructural protein (NSs)	646
TSWV NSs-R	GATGTTGTTTTCTGCTGACAT		
T7-Linker-F	GAGAATTCTAATACGACTCACTATAGGGGATCC	N/Nss	
qTSWV_N_SYBR_F	GCTTCCCACCCTTTGATTC	N	139
qTSWV_N_SYBR_R	ATAGCCAAGACAACACTGATC		
siRNA3S_F	GCGGCGTGTGAGTGAGCTTAAC	N	60
siRNA3S_RT	GTCGTATCCAGTGCAGGGTCCGAGGTATTCG- CACTGGATACGACTCTAAG		
Universal-R	GTGCAGGGTCCGAGGT		

## Data Availability

Not Applicable.
